# Ophthalmic vascular manifestations in eosinophil-associated diseases: a comprehensive analysis of 57 patients from the CEREO and EESG networks and a literature review

**DOI:** 10.3389/fimmu.2024.1379611

**Published:** 2024-04-23

**Authors:** Elisa Chapuis, Elodie Bousquet, Jean-François Viallard, Benjamin Terrier, Zahir Amoura, Veronica Batani, Antoine Brézin, Patrice Cacoub, Marco Caminati, Thibaud Chazal, Cloé Comarmond, Isabelle Durieu, Mikael Ebbo, Maximilien Grall, Emmanuel Ledoult, Laura Losappio, Irene Mattioli, Arsène Mékinian, Roberto Padoan, Francesca Regola, Jan Schroeder, Lior Seluk, Ludovic Trefond, Michael E. Wechsler, Guillaume Lefevre, Jean-Emmanuel Kahn, Pascal Sève, Matthieu Groh

**Affiliations:** ^1^ National Reference Center for Hypereosinophilic Syndromes, CEREO, Suresnes, France; ^2^ Department of Internal Medicine, Bichat Hospital, Assistance Publique-Hôpitaux de Paris, Paris, France; ^3^ Department of Ophthalmology, Lariboisière Hospital, Assistance Publique-Hôpitaux de Paris, Paris, France; ^4^ Department of Internal Medicine, Bordeaux University Hospital, Bordeaux, France; ^5^ Department of Internal Medicine, National Referral Center for Systemic and Autoimmune Diseases, Cochin Hospital, Assistance Publique-Hôpitaux de Paris, Paris, France; ^6^ Department of Internal Medicine, Autoimmune and systemic diseases, La Pitié Salpetrière Hospital, Assistance Publique-Hôpitaux de Paris, Paris, France; ^7^ Unit of Immunology, Rheumatology, Allergy and Rare Diseases, IRCCS Ospedale San Raffaele, Milan, Italy; ^8^ Department of Ophthalmology, Cochin Hospital, Assistance Publique-Hôpitaux de Paris, Paris, France; ^9^ Department of Internal Medicine and Clinical Immunology, La Pitié Salpetrière Hospital, Assistance Publique-Hôpitaux de Paris, Paris, France; ^10^ Asthma Center and Allergy Unit, Center for Hyper-Eosinophilic Dysimmune Conditions, Department of Medicine, University of Verona, Verona, Italy; ^11^ Department of Internal Medicine, Hospital Fondation Adolphe de Rothschild, Paris, France; ^12^ Department of Internal Medicine, Competence Center for Rare Autoimmune and Inflammatory Diseases, Lariboisière Hospital, Assistance Publique-Hôpitaux de Paris, Paris, France; ^13^ Department of Internal Medicine, Centre Hospitalier Universitaire Lyon Sud, Pierre-Bénite, France; ^14^ Internal Medicine Department, Hopital La Timone, APHM, Aix Marseille University, Marseille, France; ^15^ Department of Internal Medicine, CHU Rouen, Rouen, France; ^16^ Department of Internal Medicine and Clinical Immunology, CHU Lille, Lille, France; ^17^ Department of Clinical Immunology, ASST Grande Ospedale Metropolitano Niguarda, Milan, Italy; ^18^ Department of Experimental and Clinical Medicine, University of Florence, Florence, Italy; ^19^ Department of Internal Medicine, St Antoine Hospital, Assistance Publique-Hôpitaux de Paris, Paris, France; ^20^ Unit of Rheumatology, Department of Medicine DIMED, University of Padua, Padua, Italy; ^21^ Rheumatology and Clinical Immunology Unit, Department of Clinical and Experimental Sciences, ASST Spedali Civili and University of Brescia, Brescia, Italy; ^22^ Department of Medicine, National Jewish Health, Denver, CO, United States; ^23^ Department of Internal Medicine, Clermont-Ferrand University Hospital, Clermont-Ferrand, France; ^24^ Inserm, U1286 - INFINITE - Institute for Translational Research in Inflammation, University of Lille, CHU Lille, Lille, France; ^25^ Department of Internal Medicine, Ambroise Pare Hospital, Assistance Publique-Hôpitaux de Paris, Boulogne, France; ^26^ Department of Internal Medicine, Lyon University Hospital, Lyon, France; ^27^ Department of Internal Medicine, Foch Hospital, Suresnes, France

**Keywords:** eosinophilia, hypereosinophilic syndrome, eosinophilic granulomatosis with polyangiitis, retinal artery occlusion, retinal vein occlusion, retinal vasculitis, optic neuropathy

## Abstract

**Introduction:**

Eosinophils have widespread procoagulant effects. In daily practice, eosinophil-related cardiovascular toxicity consists of endomyocardial damage, eosinophilic vasculitis and arterial or venous thrombosis. Here we aim to report on the clinical features and treatment outcomes of patients with unexplained ophthalmic vascular manifestations and eosinophilia.

**Methods:**

We conducted a retrospective, multicenter, observational study and a literature review of patients with eosinophilia (≥0.5 x10^9^/L) and concomitant ophthalmic vascular manifestations independent of the underlying eosinophilic disease but with no alternative cause for ophthalmic manifestations.

**Results:**

Fifty-seven patients were included (20 from the observational study and 37 from the literature review). Ophthalmic vascular features were the initial manifestation of eosinophil-related disease in 34 (59%) patients and consisted of 29 central retinal artery occlusions, six branch retinal artery occlusions, five central retinal vein occlusions, two branch retinal vein occlusions, seven retinal vasculitides, two retinal vasospasms, 12 Purtscher’s retinopathies, 13 anterior ischemic optic neuropathies and two posterior ischemic optic neuropathies. The median [IQR] absolute eosinophil count at onset of ophthalmic vascular manifestations was 3.5 [1.7-7.8] x10^9^/L. Underlying eosinophil-related diseases included eosinophilic granulomatosis with polyangiitis (n=32), clonal hypereosinophilic syndrome (HES) (n=1), idiopathic HES (n=13), lymphocytic HES (n=2), adverse drug reactions (n=3), parasitosis (n=2), polyarteritis nodosa (n=1), IgG4-related disease (n=1), eosinophilic fasciitis (n=1) and primary sclerosing cholangitis (n=1). Other extra-ophthalmologic arterial or venous thromboses related to eosinophilia were reported in four (7%) and nine (16%) patients, respectively. Visual prognosis was poor: only eight (10%) patients achieved full recovery of ophthalmologic symptoms. After a median follow-up of 10.5 [1-18] months, one patient (3%) had a recurrence of an ophthalmic vascular manifestation, and three patients (10%) had a recurrence of other vascular symptoms (deep vein thrombosis in two and pulmonary embolism in one patient). At the time of recurrence, absolute eosinophil counts were above 0.5 x10^9^/L in all cases (n=4).

**Discussion:**

This study broadens the spectrum of vascular manifestations associated with hypereosinophilia by adding ophthalmic vascular manifestations. In patients with ophthalmological vascular manifestations and hypereosinophilia, aggressive treatment of the underlying pathology (and normalization of blood count) should be implemented.

## Introduction

Blood and/or tissue eosinophilia is a hallmark feature of multiple allergic, infectious, inflammatory, and neoplastic disorders (1). Eosinophils have widespread effects. These include the production of procoagulant phospholipids and the production of tissue factor and activated factor XII, both of which promote the genesis of thrombin (2–4). Eosinophils also release major basic protein (MBP, which contributes to platelet activation) (5, 6), eosinophilic cationic protein, eosinophil peroxidase and platelet activation factor, all of which foster thrombus formation (7, 8). According to the latest International Cooperative Working Group on Eosinophil Disorders (ICOG-Eo), both venous and/or arterial thromboses occurring in patients with absolute eosinophil counts (AEC) > 1.5 x10^9^/L are Hypereosinophilic Syndrome (HES)-defining features (9).

In daily practice, cardiovascular manifestations related to the toxicity of eosinophils mainly consist of eosinophilic myocarditis, endomyocardial fibrosis, endocardial thrombi (with potential systemic emboli) (10), venous thromboembolism (11), and eosinophilic vasculitis in patients with idiopathic HES (12, 13) Eosinophilia can also be associated with organ and/or life-threatening manifestations *e.g.*, thromboangiitis obliterans-like disease (14, 15), coronary vasospasm (16) or ischemic strokes of border zone distribution (17), all of which can have poor outcomes (18). To date, ophthalmic vascular manifestations (*e.g.* central retinal artery occlusion (CRAO), Purtscher’s retinopathy, central retinal vein occlusion (CRVO) or ischemic optic neuropathy (ION)) have seldom been reported in the setting of eosinophil-associated disorders, consisting mostly of case reports or small case series (19–22). Hence, the management of such patients is not standardized.

Here, we aim to report on the clinical picture and treatment outcomes of patients with ophthalmic vascular manifestations and eosinophilia, and ultimately to provide a data-driven practical therapeutic algorithm.

## Materials and methods

### Study design and inclusion criteria

We conducted a retrospective, multicenter, observational study. Centers involved in the French National Reference Center for HES (CEREO) and in the European Eosinophilic Granulomatosis with Polyangiitis study group as well as 1 US center of EGPA expertise (National Jewish Health, NJH) were queried to identify patients with: (i) at least one episode of ophthalmic vascular manifestation (CRAO, branch retinal artery occlusion (BRAO), CRVO, branch retinal vein occlusion (BRVO), Purtscher’s retinopathy, retinal vasculitis or ION); (ii) concomitant absolute eosinophilia count (AEC) ≥ 0.5 x10^9^/L when the ophthalmic vascular manifestation occurred. Exclusion criteria were the presence of any condition, comorbidity or concomitant treatment leading to thrombophilia (either constitutional or acquired), cardiac embolism, rhythmic heart disease, tight carotid stenosis (NASCET ≥70%) homolateral to the retinal involvement or other causes of Purtscher’s retinopathy (*e.g.* acute pancreatitis, head trauma or thrombotic microangiopath*y*), ION (*e.g.* giant cell arteritis) as well as the presence of anti-myeloperoxydase (MPO) anti-neutrophil cytoplasmic autoantibodies. A comprehensive list of exclusion criteria is provided in the **Supplementary Appendix**.

### Literature review

The PUBMED database was searched for English-language publications released up to April 2023, using the following combination of MeSH terms: (i) ‘hypereosinophilic syndrome’ (or any term referring to a condition embedded within the spectrum of clonal, reactive (including lymphocytic HES, drug-induced or paraneoplastic eosinophilia) and idiopathic HES (including single-organ and systemic eosinophil-associated diseases)); (ii) and a MeSH term referring to an ophthalmic vascular manifestation (*e.g.*, retinal artery occlusion, retinal vein occlusion, retinal vasculitis, retinal diseases, optic neuropathy). Reference lists from selected publications were screened for additional relevant studies.

### Baseline measurements

All cases were reviewed by the investigators (EC, MG) considering the entire follow-up. Using a standardized de-identified case report form, demographic (including cardiovascular and venous thromboembolism risk factors), clinical, laboratory and imaging findings at the time of the ophthalmic vascular manifestation as well as during follow-up were collected. For each patient, the underlying process underpinning blood hypereosinophilia was assessed according to the International COoperative study Group on Eosinophil disorders (ICOG-Eo) terminology (9) and thus considered as either clonal (*i.e.* neoplastic, including *FIP1L1::PDGFRA* myeloid neoplasm with eosinophilia), reactive (including all conditions that lead to the production of type 2 inflammation-related cytokines and thereby to non-clonal HE), overlapping (when embodied within the spectrum of autoimmune diseases, *e.g.* MPO ANCA-negative EGPA (23), IgG4-related disease (24), or eosinophilic fasciitis (25)), or idiopathic.

### Outcomes

For patients with ≥ 3 months of follow-up, and after exclusion of patients with single-flare eosinophilia (parasitosis and drug-induced eosinophilia), studied outcomes included visual acuity at last follow-up, the recurrence of either ophthalmic or extra-ophthalmologic vascular events, the occurrence of ophthalmic complications (*e.g.* retinal neovascularization, intravitreal hemorrhage or neovascular glaucoma), and death. Full ophthalmic recovery was defined as the resolution of ophthalmologic symptoms (full correction of visual acuity, visual field normalization, no recurrence of transient monocular blindness). Partial recovery was defined as partial improvement in visual acuity and/or visual field.

### Statistical analysis

Patient characteristics are reported as median [interquartile] ([IQR]) and frequency (percentage) for continuous and categorical variables, respectively. Visual acuity was converted into the log of the minimum angle of resolution (logMAR). Patient subsets were differentiated based on the subtype of vascular manifestation (arterial involvement *vs.* venous thrombosis *vs.* Purtscher’s retinopathy). Visual outcomes were compared using the Chi-squared test and continuous variables were compared using the Kruskal Wallis test.

### Ethical and regulatory considerations

All methods were carried out in accordance with relevant guidelines and regulations (*i.e.* the Good Clinical Practice protocol, the Declaration of Helsinki principles and the MR004 French legislation regarding observational retrospective studies) and this study was approved by the independent ethics committee of Foch Hospital (IRB00012437, approval number 23-03-04).

## Results

### Patient identification and baseline characteristics

**Figure 1:**
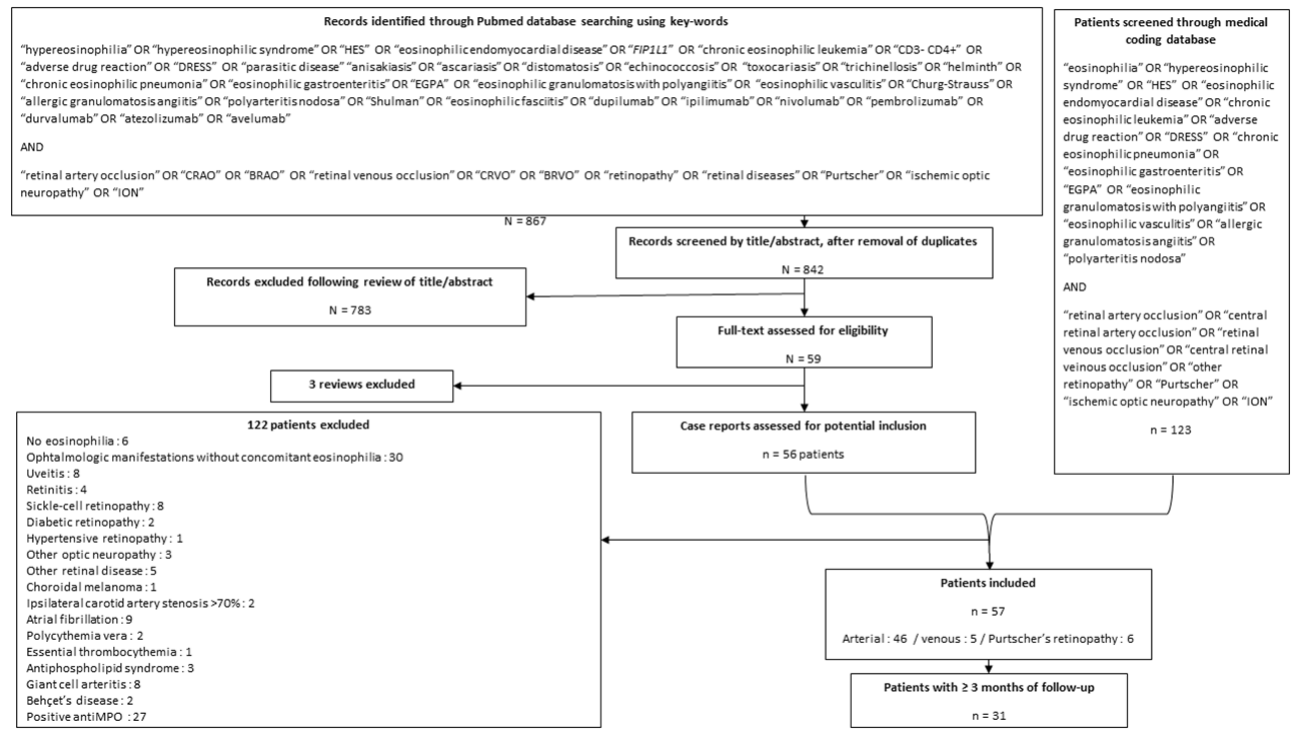
Flow-chart showing the search strategy and inclusion/exclusion criteria for the study population.

One hundred and twenty-three patients were screened through the CEREO, EESG and NJH databases, and 55 case reports (corresponding to 56 patients) treated for eosinophilia and concomitant ophthalmic vascular manifestations were identified through the literature review. Overall, 57 patients fulfilled inclusion criteria (20 from the observational study and 37 from the literature review, **Figure 1**). Their main characteristics are reported in **Table 1**. Thirty-two (56%) were male and their median age at ophthalmic manifestation onset was 54 [44-65] years. Thirty-six (58%) had at least one cardiovascular risk factor, three (5%) had a prior history of cardiovascular disease and four (7%) had a prior history of venous thromboembolism. Among the 55 patients with available data, only one (2%) developed ophthalmic vascular involvement despite ongoing treatment with both antiplatelets and anticoagulants.

**Table 1:** Baseline demographic, clinical, biological and treatment features of patients with ophtalmic vascular manifestations and eosinophilia.

	All patients (n=57, eyes 78)	Arterial involvement (n=46, eyes 59)	Venous involvement (n=5, eyes 7)	Purtscher's retinopathy (n=6, eyes 12)
**Demographics**				
Sex, female	25 (44)	18 (39)	3 (60)	4 (67)
Age, years	54 [44-65]	56 [46-66]	53 [45-56]	39 [32-45]
Cardiovascular risk factors				
Age > 50years (man) or > 60years (woman)	30 (53)	27 (59)	2 (40)	1 (17)
Active smoker	3/38 (8)	3/29 (10)	0 (0)	0 (0)
Hypertension	13/38 (34)	9/29 (31)	2 (40)	2 (33)
Diabetes	6/38 (16)	3/29 (10)	2 (40)	1 (17)
Dyslipidemia	1/38 (3)	1/39 (3)	0	0
Number of cardiovascular risk factors	1 [0-1]	1 [0-1]	1 [1-1]	0 [0-0]
Prior history of cardiovascular disease	3 (5)	3 (7)	0	0
Prior history of venous thromboembolic disease	4 (7)	2 (4)	1 (20)	1 (20)
**Ophthalmological findings**				
Bilateral involvement	21 (37)	13 (28)	2 (40)	6 (100)
Ophthalmological symptoms				
Visual acuity loss	71 (91)	52 (88)	7 (86)	12 (100)
Visual field abnormalities	9 (12)	7 (12)	0 (0)	2 (20)
Transient monocular blindness	13 (17)	12 (20)	1 (14)	0
Visual acuity (logmar)	1.7 [0.5-2.3]	2 [0.8-2.3]	0.45 [01-0.88]	1.7 [0.5-1.7]
Fundus abnormalities	64/64 (100)	47/47 (100)	5/5 (100)	12/12 (100)
Fluorescein angiography abnormalities	37/39 (95)	27/29 (93)	2/2 (100)	8/8 (100)
Optical Coherence Tomography abnormalities	14/18 (78)	5/9 (56)	3/3 (100)	616 (100)
Visual field defects	14/17 (82)	12/15 (80)	0/0	2/2 (100)
Ophtalmological diagnoses				
CRAO	29	29	–	–
BRAO	6	6	–	–
AION	13	13	–	–
PION	2	2	–	–
Vasculitis	7	7	–	–
Retinal vasospasm	2	2	–	–
Purtscher 's retinopathy	10	–	–	12
CRVO	5	–	5	–
BRVO	2	–	2	–
**Eosinophilic workup**				
Prior eosinophil-associated disorder	23 (41)	17 (37)	4 (80)	2 (33)
Main conditions underlying eosinophilia				
Clonal HES	1 (2)	1 (2)	0	0
Idiopathic HES	13 (23)	7 (15)	2 (40)	4 (67)
Lymphocytic HES	2 (4)	2 (4)	0	0
EGPA	32 (57)	30 (65)	2 (40)	0
Polyarteritis nodosa	1 (2)	1 (2)	0	0
Parasitosis	2 (4)	2 (4)	0	0
Adverse drug reaction	3 (5)	1 (2)	0	2 (33)
Others	3 (5)	2 (4)	1 (20)	0
Extra-ophthalmological eosinophil- related organ involvement	48 (86)	39 (88)	4 (80)	5 (100)
n of other organs involved	2 [1-3]	2 [1-3]	2 [2-3]	3 [3-4]
Arterial thrombosis	4 (7)	4 (9)	0	0
Venous thrombosis	9 (16)	6 (13)	2 (40)	1 (20)
Heart	4 (7)	4 (9)	0	0
Lung	16 (29)	11 (24)	3 (60)	2 (40)
Skin	28 (50)	22 (48)	2 (40)	4 (80)
Gastrointestinal tract	2 (4)	2 (4)	0	0
ENT	17 (30)	16 (35)	1 (20)	0
Central nervous system	4 (7)	2 (4)	1 (20)	1 (20)
Peripheral nervous system	23 (41)	22 (48)	1 (20)	0
Kidney	3 (5)	1 (2)	1 (20)	1 (20)
Musculoskeletal	12 (21)	8 (17)	2 (40)	2 (40)
Effusion of serous cavities	2 (4)	1 (2)	0	1 (20)
Liver and biliary tract	4 (7)	2 (4)	1 (20)	1 (20)
Laboratory findings				
AEC at admission for ophthalmologic event, x 10^9/L	3.5 [1.7-6.8]	3 [1.6-4.7]	3 [1.8-5.9]	5 [5-30]
Maximum AEC , x 10^9/L	4.6 [2-8.9]	4.2 [2-8.1]	3 [1.8-5.9]	5 [5-30]
ANCA positive	8/36 (22)	7/25 (28)	1/3 (33)	0
CRP > 40mg/L	7/32 (22)	7/28 (25)	0/3 (0)	1/1 (100)
Elevated tryptase	0/11 (0)	0/9	0/1	0/1
Elevated IgE	9/12 (75)	8/10 (80)	1/1 (100)	0/1 (0)
**Treatments**				
After the ophthalmic event				
Glucocorticoids	45 (79)	39 (85)	0	6 (100)
Immunosuppressants	12 (21)	12 (26)	0	0
Intravenous immunoglobulins	1 (2)	1 (2)	0	0
Plasma exchanges	2 (4)	2 (4)	0	0
Antiparasitic therapy	1 (2)	1 (2)	0	0
Fibrinolysis	5 (9)	5 (11)	0	0
Ocular hypotensive therapy	4 (7)	4 (9)	0	0
During the entire follow-up				
Glucocorticoids	48 (84)	41 (89)	1 (20)	6 (100)
Immunosuppressants	20 (36)	19 (41)	1 (20)	0
Anti -IL5/IL5R	7 (13)	6 (13)	1 (20)	0
Imatinib	2 (4)	2 (4)	0	0
Hydroxyurea	2 (4)	2 (4)	0	0
Infiiximab	1 (2)	1 (2)	0	0
Rituximab	3 (5)	2 (4)	1 (20)	0
Plasma exchanges	2 (4)	2 (4)	0	0
Antiparasitic therapy	3 (5)	3 (7)	0	0
Intravenous immunoglobulins	1 (2)	1 (2)	0	0
Antiplatelets	21 (38)	20 (43)	1 (20)	0
Anticoagulants	9 (16)	7 (15)	2 (40)	0

Data are reported as no. (%) or median [IQR], unless otherwise specified.

AEC, absolute eosinophil count; AION, anterior ischemic optic neuropathy; BRAO, branch retinal artery occlusion; BRVO, branch retinal vein occlusion; CRAO, central retinal artery occlusion; CRVO, central retinal vein occlusion; EGPA, eosinophilic granulomatosis with polyangiitis; ENT, ear nose and throat; HES, hypereosinophilic syndrome; PION, posterior ischemic optic neuropathy.

Ophthalmologic symptoms were the initial eosinophil-related organ involvement in 34 (59%) patients, while 23 (41%) had already been diagnosed with a prior eosinophil-associated disease, including 17 (30%) who were currently treated with systemic corticosteroids at the time of ophthalmic vascular involvement and four (7%) who had prior eosinophil-related venous thromboembolism. Among the seven patients with available data, the median delay between eosinophil-related first manifestation and ophthalmic symptoms was 36 [15-96] months. The median AEC at onset of ophthalmic vascular manifestation was 3.5 [1.7-6.8] x10^9^/L. Among the 40 patients (70%) with available data, the median peak AEC during follow-up was 4.6 [2-8.9] x10^9^/L, including 37 (92%) patients with hypereosinophilia > 1.5 x10^9^/L (*i.e.* the HES-defining threshold). Among the 48 patients with extra-ophthalmologic eosinophil-related organ involvement, the median number of organs involved was two [1-3], consisting mostly of peripheral nervous system (n=23), skin (n=28), lung (n=16), ENT (n=17) and musculoskeletal (n=12) manifestations. Of note, four (7%) patients reported features of arterial thrombosis (ischemic stroke n=2, acute coronary syndrome and gastrointestinal tract ischemia, a single patient each), nine (16%) of venous thromboembolism (pulmonary embolism n=4, lower limb deep venous thrombosis n=3, inferior vena cava thrombosis, pulmonary vein thrombosis, a single patient each), and four (7%) had eosinophilic myocarditis. Among the 13 thrombotic manifestations, seven occurred concomitantly with ophthalmic vascular manifestations. Eosinophilic granulomatosis with polyangiitis (EGPA, formerly Churg-Strauss syndrome) was the leading cause driving eosinophilia (n=32, 57%). Other associated conditions included *STAT5A*-associated chronic eosinophilic leukemia (n=1, 2%), lymphocytic (n=2, 4%), idiopathic (n=13, 23%), reactive (n=5, 7% including three drug adverse reactions and two parasitosis) HES as well as overlapping diseases (n=4, 7%, consisting of polyarteritis nodosa, IgG4-related disease, sclerosing cholangitis and eosinophilic fasciitis, a single patient each).

### Ophthalmological findings

**Figure 2:**
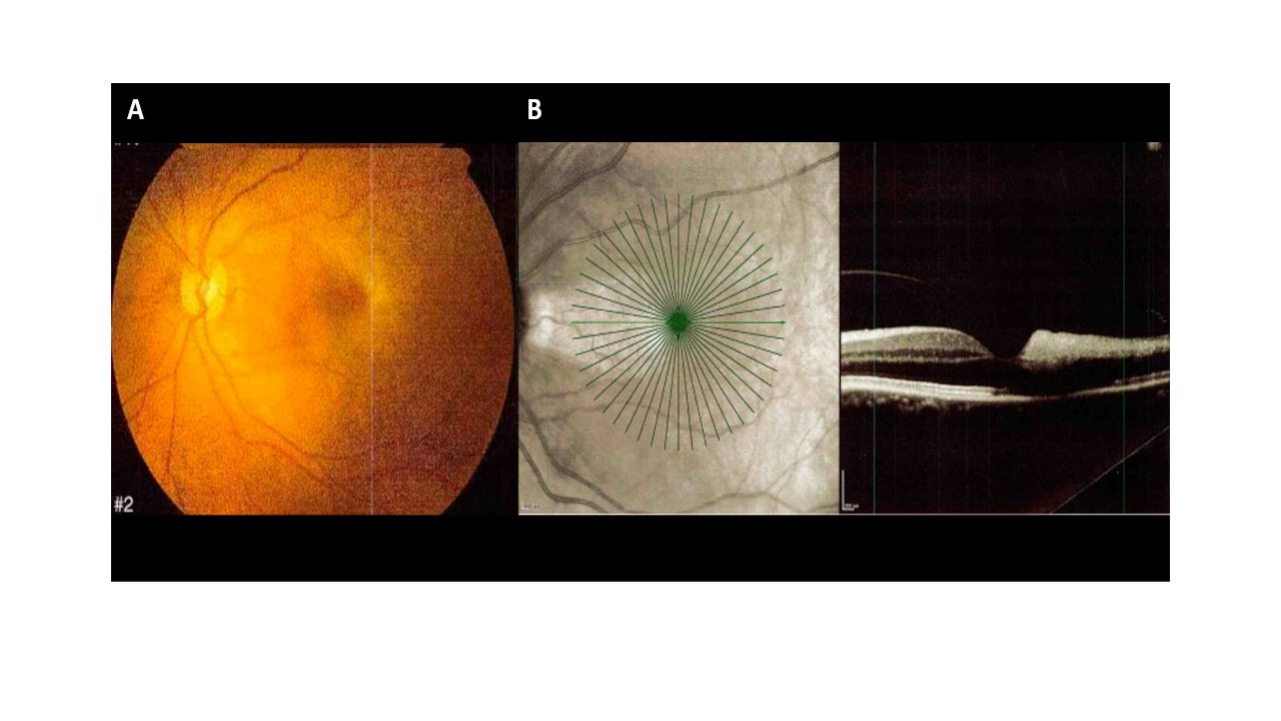
Color fundus photography and spectral domain optical coherence tomography (SD-OCT) of a 43-year-old woman with eosinophilia and central retinal artery occlusion (CRAO) in the left eye. **(A)** Color fundus photography illustrates a retinal whitening of the posterior pole indicative of a CRAO with preservation of the cilioretinal artery. **(B)** SD-OCT shows hyperreflectivity in the temporal middle and inner retinal layer hyperreflectivity consistent with CRAO and cilioretinal artery sparing.

Ocular involvement was bilateral in 21 patients (37%), and in the remaining patients there was no predominant eye involved. Ophthalmic vascular manifestations included CRAO (n= 24 patients, 29 eyes), BRAO (n= 5 patients, 6 eyes), CRVO (n= 3 patients, 5 eyes), BRVO (n= 2 patients, 2 eyes), retinal vasculitis (n= 5 patients, 7 eyes), retinal vasospasm evidenced upon fluorescein angiography (one patient with bilateral involvement), Purtscher’s retinopathy (n=6 patients, 12 eyes), anterior ischemic optic neuropathy (n= 10 patients, 13 eyes) and posterior ischemic optic neuropathy (n=2 patients with unilateral involvement). Ocular symptoms (present in all patients but one) consisted of vision loss (53 patients, 71 eyes), transient monocular blindness (11 patients, 13 eyes) and visual field abnormalities (7 patients, 9 eyes). The median visual acuity at diagnosis was 1.7 [0.5-2.3] logmar. In all eyes with available data (n=64 eyes), fundus examination was always abnormal (fundus data of the 2 patients with posterior ischemic optic neuropathy were not available). Among the 39 fluorescein angiographies that were performed, imaging findings were abnormal and concordant with the clinical diagnoses suspected upon fundus examination in 37 (95%) cases. **Figure 2** illustrates the retinal imaging finding of a patient with CRAO in the left eye. Among the 28 patients with imaging of supra-aortic arteries, 8 (29%) had evidence of mild (NASCET < 70%) ipsilateral carotid artery atheroma. Of note, among the 22 patients with available concomitant brain imaging (cerebral MRI or injected TDM), cerebral ischemic events were reported in two (5%) cases.

### Treatment regimens

At the acute phase, forty-five (79%) patients received systemic corticosteroids (oral corticosteroids, n=23; intravenous corticosteroids n=22; starting doses ranging from 10mg to 1mg/kg/day of prednisone or equivalent before gradual tapering). Twelve (21%) patients received immunosuppressants, including cyclophosphamide (n=8), azathioprine (n=3) and methotrexate (n=1). Fibrinolytic agents and ocular hypotensive therapy were prescribed in five (9%) and four (7%) patients, respectively, while nine (16%) and 21 (38%) patients received long-term anticoagulants or antiplatelets, respectively. Other treatments consisted of antiparasitic drugs (n=1), plasma exchanges (n=2) and intravenous immunoglobulins (n=1).

During follow-up, other treatments included imatinib (n=2), hydroxyurea (n=2) and infliximab (n=1).

### Outcomes

Full details of patients' outcomes are provided in **Table 2**. Among the 50 (88%) patients with available follow-up data (including 30 with more than three months of follow-up), the median follow-up after the initial ophthalmic vascular manifestation was 10.5 [1-18] months. One (3%) patient had a recurrence of ophthalmic vascular manifestation (CRVO) and three (10%) patients had another vascular event (lower limb deep vein thrombosis n=2, pulmonary embolism n=1). In all cases, AEC was above 0.5 x10^9^/L at time of recurrence. At last follow-up, only six patients (12%) achieved full recovery. Sixteen patients (32%) achieved partial recovery, 23 patients (46%) stabilized once under treatment, while the condition of the remaining five patients (10%) worsened despite therapy. By comparison with patients with arterial involvement, the rate of ophthalmic recovery was higher in patients with either Purtscher’s retinopathy or venous involvement (recovery achieved in 8/10 eyes with Purtscher’s retinopathy and in 4/5 eyes with venous involvement *vs* 18/53 eyes with arterial involvement, p=0.019). Patients with venous involvement or Purtscher’s retinopathy had a better visual acuity at last follow-up than patients with arterial involvement (median logmar 0 [0-0], 1 [0-2.3] and 0.5 [0.2-0.8] for patients with venous, arterial involvement and Purtscher’s retinopathy respectively; p= 0.038). Of note, the levels of baseline absolute eosinophil counts did not correlate with long-term visual outcomes, and there were no significant differences regarding the final visual acuities of patients who received antiplatelets vs. anticoagulants as well as in patients who received only corticosteroids vs. those who received both corticosteroids and immunosuppressants (data not shown).

**Table 2:** Outcomes of patients with ophthalmic vascular manifestations and eosinophilia.

	All patients (n=57, 78 eyes)	Arterial involvement (n=46, 59 eyes)	Venous involvement (n=5, 7 eyes)	Purtscher's retinopathy (n=6, 12 eyes)
**Follow-up time (month)**	10.5 [1-18]	11 [1-31]	8.5 [3.8-12]	6 [1-12]
**Visual status at last follow-up (eyes)**				
Worsening	8 (10)	8 (14)	0	0
Stability	30 (38)	27 (46)	1 (20)	2 (20)
Partial recovery	22 (28)	14 (24)	0	8 (80)
Full recovery	8 (10)	4 (7)	4 (80)	0
Visual acuity at last follow-up (logmar)	0.5 [0-2]	1 [0-2.3]	0 [0-0]	0.5 [0.2-0.8]
Ophthalmological complications	3 (10)	3 (13)	0	0
Vascular ophthalmologic recurrence	1 (3)	0	1 (33)	0
**Other outcomes (n = 30 patients)**				
Other vascular event	3 (10)	3 (13)	0	0
Death	4 (7)	3 (7)	0	1 (20)

Data are reported as no. (%) or median [IQR].

Three (10%) patients developed retinal neovascularization with subsequent intravitreal hemorrhage during follow-up. During follow-up, four patients died of pulmonary infection, MRSA-induced septic shock, endomyocardial fibrosis and hepatitis (a single patient each). Both patients who died of sepsis were treated by systemic corticosteroids (in addition to methotrexate or mepolizumab, a single patient each).

### Focus on patients with EGPA

Thirty-two (57%) patients fulfilled MIRRA and/or ACR criteria for EGPA (all without MPO-ANCA). In the latter patients, ophthalmic vascular manifestations consisted of CRAO (17 patients, 21 eyes), BRAO (a single patient with unilateral involvement), AION (8 patients, 11 eyes), PION (a single patient with unilateral involvement), retinal vasculitis (3 patients, 4 eyes), CRVO (a single patient with bilateral involvement) and BRVO (a single patient with unilateral involvement). It was noteworthy that no patient with EGPA had Purtscher’s retinopathy. Extra-ophthalmic vascular manifestations included both eosinophilic-driven (*e.g.* eosinophilc myocarditis or central nervous system involvement, a single patient each) and vasculitic (*e.g.* mononeuritis multiplex n=18 patients and purpura, n=12 patients) manifestations. Among the 19 patients with available data, only five (26%) had mild elevation of C-reactive protein (CRP) levels (<40mg/L).

The visual prognosis of EGPA patients was poor and the median final visual acuity was 2 [0.1-2.4] logmar. Among the fourteen patients with at least three months of follow-up, only one patient recovered completely and three achieved partial recovery, whereas two patients worsened and eight had a stability of their visual acuity. Moreover, three patients (21%) had a new vascular event during follow-up (lower limb deep vein thrombosis, n=2; pulmonary embolism, n=1). None of the 32 EGPA patients died during follow-up.

## Discussion

Recent advances have led to the better understanding of the mechanisms driving the pro-coagulant effects of eosinophils (26), and reported cases of arterial and venous thrombotic manifestations related to eosinophilia have increased (11, 15–17, 19). Likewise, the spectrum of eosinophilia-related cardiovascular toxicity has now broadened beyond the scope of eosinophilic cardiomyopathy (10). Some studies have recently highlighted peculiar phenotypes *e.g.*, thromboangiitis obliterans-like disease (14), eosinophilia-associated coronary vasospasm (16) or ischemic strokes of border zone distribution (17). Here, we shed further light on the diversity of eosinophil-induced vascular symptoms and report on various ophthalmic vascular manifestations occurring within the full-spectrum of eosinophil-related diseases, either as first disease manifestation or during follow-up.

Intracardiac thrombus and peripheral arterial emboli were the main features reported in the review of HES-related cardiovascular manifestations reported by Ogbogu et al (10). Likewise, in the main series of patients with HES, ophthalmic vascular manifestations have rarely been reported (19, 27, 28), and mostly consist of case reports or small case series (20–22). In their 2019 review of 189 patients with idiopathic eosinophilic vasculitis, Lefevre et al. reported on only one case of CRAO and one case of retinal vasculitis (12). Among 151 patients with *FIP1L1::PDGFRA*-related HES, only one case of CRAO was reported (19) and in another series of 26 patients with CD3^-^CD4^+^ lymphocytic HES, none presented with ophthalmic symptoms (27). Dupilumab-induced Purtscher’s retinopathy with eosinophilia reported herein is in line with dupilumab-induced eosinophilic vasculitis that our group has previously reported (29). EGPA is the only eosinophil-associated disease for which ophthalmic vascular manifestations have been more extensively depicted, with predominant arterial involvement and poor visual prognosis despite treatment with corticosteroids (30, 31).

Here, we report on a wide variety of ophthalmic vascular manifestations related to eosinophil-associated disorders, that clustered into three main clinical pictures (*i.e.* arterial or venous retinal occlusions and Purtscher’s retinopathy), with one in three patients having bilateral involvement. There was no clear correlation between the clinical picture and underlying eosinophil-related diseases, supporting the fact that eosinophilia, whatever its cause, can lead to ophthalmologic vascular toxicity. Nevertheless, both clonal and lymphocytic HES were rare (one and two patients respectively), and EGPA was never reported in the setting of Purtscher’s retinopathy. Strikingly, other (and most often concomitant) extra-ophthalmologic vascular manifestations related to eosinophilia were reported in up to 15% of patients, including organ or life-threatening events *e.g.*, ischemic strokes, acute coronary syndrome, gastrointestinal tract ischemia, or inferior vena cava thrombosis. Although skin, lung and gastrointestinal symptoms are the most frequent manifestations of HES, the latter is a multifaceted disease and some patients have prominent vascular manifestations, including catastrophic antiphospholipid syndrome-like presentation (11). As expected (32–35), the visual prognosis was poor (with only six patients achieving full recovery and significant loss of visual acuity at last follow-up), especially in patients with ION or CRAO.

In this series, patients had few cardiovascular risk factors and no major risk factor for venous thromboembolism, suggesting that their ophthalmic manifestations indeed were the consequence of eosinophil-related toxicity. There is strong evidence substantiating the procoagulant effects of eosinophils and their direct toxicity on the vascular endothelium. First, injury-induced venous thrombosis is drastically reduced in both eosinophil-deficient and eosinophil-depleted mice (2). Moreover, eosinophils are potent producers of tissue factor (2–4), can produce procoagulant phospholipids and activate factor XII, which both stimulate the intrinsic coagulation pathway (2). Eosinophils also release major basic protein (MBP), a cationic protein that binds to thrombomodulin and thereby impairs its anticoagulant effects (5, 6). Likewise, the discharge of cytotoxic granules and pro-inflammatory mediators increases vascular permeability and induce endothelial damage, both of which contribute to a procoagulant state (26). Eosinophil extracellular DNA traps also promote platelet activation (36, 37). Lastly, since EGPA accounted for more than half of underlying eosinophil-related diseases, it is likely that underlying vasculitis also contributes to the clinical picture. Nevertheless, patients tested positive for MPO-ANCA were excluded from the study, and only a minority of patients had histologic evidence (or strong clinical surrogates) of vasculitis. Moreover, inflammatory markers tended to be low, suggesting a direct pathogenic role of eosinophils rather than systemic vasculitis (12).

As eosinophils seem to have a prominent role in the genesis of ophthalmic vascular manifestations, prompt initiation of eosinophil-targeted treatments is advisable to curb the deleterious pathophysiological process and to prevent the advent of other manifestations related to eosinophilia-related vascular toxicity. Corticosteroids are the cornerstone of the management of most eosinophilia-associated diseases (38–40). Here, 84% of patients received corticosteroids, which are rapidly effective in most cases (91%) except in a very limited number of well-defined conditions, including drug-hypersensitivity, clonal HES, and paraneoplastic eosinophilia (18). The use of corticosteroids has also seldom been reported in Purtscher’s retinopathy or CRVO, as evidence is lacking (33, 35). Although more than one third of patients received immunosuppressants, it should be emphasized that, in both HES and EGPA, there is no evidence that the adjunction of either cytotoxic drugs or anti-interleukin 5 biologics to corticosteroids is superior to corticosteroids alone at the acute phase (38, 40). Nevertheless, as mepolizumab has demonstrated clinical efficacy and substantial steroid-sparing effect in both EGPA and HES, it is likely that such treatment is beneficial on the long run in patients with high dose steroid dependency and/or steroid-related side effects (41–43).

**Figure 3:**
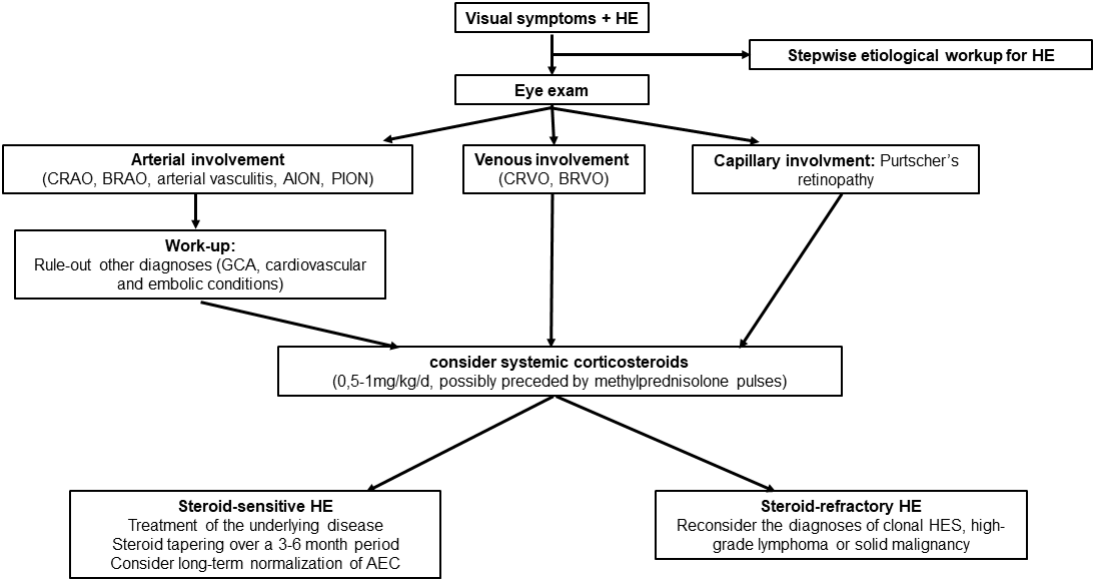
Suggested algorithm for the management of eosinophil-related ophthalmic vascular manifestations. HE, hypereosinophilia; CRAO, central retinal artery occlusion; BRAO, branch retinal artery occlusion; AION, anterior ischemic optic neuropathy; PION, posterior ischemic optic neuropathy; CRVO, central retinal vein occlusion; BRVO, branch retinal vein occlusion; GCA, giant cell arteritis.

CRAO presents a significant challenge in ophthalmology, as it often leads to irreversible retinal damage within four hours of the retinal artery occlusion. Unfortunately, there is currently no established treatment or evidence-based therapy available for non-arteritic CRAO. Numerous approaches have been attempted with the goal of dislodging emboli and enhancing retinal blood flow through various procedures, such as ocular massage, intraocular pressure reduction, isovolemic hemodilution, anticoagulation, and intraarterial fibrinolysis. These interventions have yielded poor visual outcomes (44, 45). Conversely, a strict management of cardiovascular risk factors is encouraged to manage CRVO (41), and there is no standard of treatment for Purtscher’s retinopathy (35). Overall, both anticoagulants (at the acute phase) and antiplatelets (on the long run) were also frequently prescribed. As the only recurrence of ophthalmic vascular manifestation occurred in a patient with persistent eosinophilia, long-term normalization of AEC (thanks to treatment of the underlying condition) is advisable and is likely to prevent the recurrence of vascular manifestations. Of note is that in venous thromboembolism related to eosinophilia, we have previously demonstrated that anticoagulants could be safely withdrawn once complete hematological response was obtained in the long run. A suggested algorithm for the management of eosinophil-associated vascular ophthalmic involvement is provided in **Figure 3**.

This study has several drawbacks, including missing data and lack of standardized management within centers. Next, little follow-up data was available from the cases retrieved from the literature review, which possibly led to an underestimation of the risk of recurrence. Lastly, given the retrospective design of the study, we were unable to assess whether, besides AEC, other biological parameters (including markers of eosinophil activation and degranulation) could also correlate with outcomes.

Regardless of these limitations, this study – the first longitudinal analysis dedicated to ophthalmic vascular manifestations occurring during eosinophil-related diseases – emphasizes the fact that eosinophilia (whatever the underlying disease) can lead to ophthalmic vascular toxicity. It provides useful data for both ophthalmologists and physicians involved in the field of eosinophil-related disorders and suggests that, in a subset of patients with otherwise unexplained ophthalmic vascular manifestation and eosinophilia, long-term normalization of AEC is advisable to prevent the recurrence of vascular manifestations. Further large-scale studies are needed to confirm these preliminary findings, and collaborative endeavors are welcome.

## Data availability statement

The original contributions presented in the study are included in the article/supplementary material. Further inquiries can be directed to the corresponding author.

## Ethics statement

The studies involving humans were approved by independent ethics committee of Foch Hospital (IRB00012437, approval number 23-03-04). The studies were conducted in accordance with the local legislation and institutional requirements. The participants provided their written informed consent to participate in this study. Written informed consent was obtained from the individual(s) for the publication of any potentially identifiable images or data included in this article.

## Author contributions

EC: Formal analysis, Investigation, Writing – original draft, Writing – review & editing, Conceptualization, Methodology. EB: Writing – review & editing, Conceptualization, Data curation, Investigation. JV: Data curation, Writing – review & editing. BT: Data curation, Writing – review & editing. ZA: Data curation, Writing – review & editing. VB: Data curation, Writing – review & editing. AB: Data curation, Writing – review & editing. PC: Visualization, Writing – review & editing, Data curation. MC: Data curation, Writing – review & editing, Validation. TC: Writing – review & editing. CC: Writing – review & editing. ID: Data curation, Writing – review & editing. ME: Data curation, Writing – review & editing. MGra: Data curation, Writing – review & editing. EL: Data curation, Writing – review & editing. LL: Data curation, Writing – review & editing. IM: Data curation, Writing – review & editing. AM: Data curation, Writing – review & editing. RP: Data curation, Writing – review & editing. FR: Data curation, Writing – review & editing. JS: Data curation, Writing – review & editing. LS: Data curation, Writing – review & editing. LT: Data curation, Writing – review & editing. MW: Data curation, Writing – review & editing. GL: Data curation, Writing – review & editing. JK: Data curation, Writing – review & editing. PS: Data curation, Writing – review & editing. MGro: Conceptualization, Data curation, Investigation, Methodology, Supervision, Writing – original draft, Writing – review & editing.
