# Supplementary material for: Ophthalmic vascular manifestations in eosinophil-associated diseases: a comprehensive analysis of 57 patients from the CEREO and EESG networks and a literature review
Source: Front Immunol. 2024 Apr 23;15:1379611. doi: 10.3389/fimmu.2024.1379611 (PMC11078014; doi:10.3389/fimmu.2024.1379611)
Supplement: Supplementary file 1 [file DataSheet_1.pdf]

International Classification of Diseases codes (tenth revision) used for the screening of patients:

1. Codes related to retinal vascular pathology:

- Central retinal artery occlusion H341
- Other retinal artery occlusions H342
- Retinal vascular occlusions H343
- Central retinal vein occlusion H348
- Ischemic optic neuropathy (ION) H470
- Other retinopathies and retinal vascular alterations H350

AND

2. Codes related to hypereosinophilia:

- Eosinophilia D72.1
- Hypereosinophilic syndrome D47.5
- Eosinophilic endomyocardial disease I42.3
- Chronic eosinophilic pneumonia J82
- Chronic eosinophilic leukemias D47.5
- Eosinophilic gastroenteritis K52.8
- Polyarteritis nodosa M30.0
- Eosinophilic granulomatosis with polyangiitis (EGPA, formerly Churg-Strauss syndrome) M30.1
- Allergic granulomatous angiitis M30.1
- Adverse drug reaction Y579
- DRESS T887

Exclusion criteria:

1. Major constitutional thrombophilia:

- Homozygous factor II mutation
- Homozygous factor V mutation
- Double heterozygosity for factors II and V
- Hypofibrinogenemia < 1 g/L
- Protein S deficiency < 40% in the absence of treatment with vitamin K antagonists (VKA)
- Protein C deficiency < 60% in the absence of treatment with VKA
- Antithrombin III deficiency < 80%
- Hyperhomocysteinemia ≥ 30 µmol/L

2. Acquired thrombophilia:

- Antiphospholipid syndrome according to Sydney criteria
- Behçet's disease according to the International Criteria for Behçet’s Disease
- Active inflammatory bowel disease
- Nephrotic syndrome with albumin < 20g/L
- Solid cancer under treatment

- Hodgkin's lymphoma
- T-cell lymphoma excluding squamous cell lymphoma
- Acute leukemia
- Thrombocytosis with platelet count $\geq x10^{12}/L$
- Polycythemia (hemoglobin ≥ 16.5 g/dL in men or ≥ 16 g/dL in women)

3. Other transient increased risks of thrombosis:

- Surgery in the previous month
- Third trimester of pregnancy
- Post-partum (up to 6 weeks after delivery)
- Birth control with cyproterone acetate-based estrogen-progestin pill
- Initiation of contraception with estrogen-progestin pill in the 6 months preceding the ophthalmic manifestation
- Initiation of oral postmenopausal hormone replacement therapy within 2 years prior to the ophthalmic manifestation
- Any hormonal treatment as part of a medically assisted reproduction procedure
- Treatment with erythropoietin
- Treatment with thrombopoietin receptor agonists
- Treatment with lenalidomide or thalidomide

4. Presence of anti-myeloperoxydase (MPO) anti-neutrophil cytoplasmic antibodies (ANCA).

5. For arterial involvement specifically:

- Emboligenic cardiopathy, *e.g.* rhythmic heart disease (without effective anticoagulation), intracavitary thrombus, left ventricular aneurysm, grade III or IV mitral stenosis or infectious endocarditis
- Tight carotid stenosis (NASCET ≥ 70%) homolateral to the retinal involvement

6. For Purtscher’s retinopathy specifically:

- Acute pancreatitis
- Head or chest trauma
- Thrombotic microangiopathy

7. Specifically, for ION

- Giant cell arteritis
